# Vanillin production by biotransformation of phenolic compounds in fungus, *Aspergillus luchuensis*

**DOI:** 10.1186/s13568-018-0569-4

**Published:** 2018-03-13

**Authors:** Junsei Taira, Rin Toyoshima, Nana Ameku, Akira Iguchi, Yasutomo Tamaki

**Affiliations:** Department of Bioresource Technology, Okinawa National College of Technology, 905 Henoko, Nago, Okinawa 905-2192 Japan

**Keywords:** *Aspergillus* species, Phenolic compound, Biotransformation, Vanillin, Ferulic acid, LC/MS

## Abstract

Vanillin is valuable and popular flavor used in foods and cosmetics. Many bacteria species have the ability to decarboxylate substituted cinnamic acids in order to form vanillin. However, the phenolic biotransformation including vanillin production in a common fungus, the *Aspergillus luchuensis*, which is used in distilled beverages, has not yet been clarified. This study focused on elucidating the vanillin production due to phenolic biotransformation in *A. luchuensis* during fermentation. The phenolic metabolites were extracted by a solid phase column and they were determined using on LC/MS and LC/MS/MS in a selective ion mode. As a result, ferulic acid, vanillin and vanillic acid, were detected in the rice koji fermentationed by *A. luchuensis* and also fermentated with yeast. In addition, the accurate molecular formula of vanillin glucoside (C_14_H_17_O_8_, 313.0927, (M-H)^−^ and its production ions was also determined by HRESI-mass spectrometry. Based on the results including the phenolic metabolites and related genes found in *A. luchuensis* genome, this study proposed the vanillin production mechanism due to the side chain cleavage of ferulic acid through Coenzyme A (CoA) and feruloyl-CoA hydratase/lyase, to form vanillin and acetyl-COA. In this study, another possible vanillin production pathway also was proposed due to the neutral hexose hydrolysis of vanillin glucoside. The subsequent dehydrogenation of vanillin produced vanillic acid. In addition, vanillin was detected in the distilled alcohol indicating its contribution to the aroma profile of beverages. It has been unknown that the vanillin in the distilled solution is derived from the vanillin produced during rice-koji and/or moromi mash fermentations.

**Electronic supplementary material:**

The online version of this article (10.1186/s13568-018-0569-4) contains supplementary material, which is available to authorized users.

## Introduction

Vanillin is an aromatic flavor compound which is frequently used in foods and cosmetics. Its production process by biotransformation has been intensively studied. Many bacteria species have the ability to decarboxylate substituted cinnamic acids, such as ferulic acid, eugenol, isoeugenol and *p*-coumaric acid, which are converted to useful aromatic chemicals, such as 4-vinyl guaiacol and 4-ethyl phenol (Priefert et al. 2001; Rabenhorst 1996; Narbad and Gasson 1998; Shimoni et al. 2000; Masai et al. 2002). Particularly, ferulic acid biocatalytically converted to produce vanillin, thus most attention has focused on the bioconversions of ferulic acid (Beek and Priest 2000; Huang et al. 1993). Two types of ferulic acid side chain cleavages have been reported for a number of microorganisms (Rosazza et al. 1995). One type is catalyzed by nonoxidative decarboxylase, which eliminates one carbon from the ferulic acid side chain, resulting in the formation of 4-vinyl guaiacol. The other type is characterized by the elimination of two carbons from the ferulic acid side chain, and this latter reaction can produce vanillin, a valuable flavor compound. The role of lactic acid bacteria in the conversion of phenolic compounds to vanillin has been elucidated during the brewing of whisky and wine (Rosazza et al. 1995; Cavin et al. 1993; Bloem et al. 2007). East Asian countries, such as Japan, Philippine, China and Thailand have been using a common fungus for brewing. The Japanese spirit, *shochu*, is a traditional distilled beverage that uses a common fungus, the *Aspergillus* species, with the raw materials, such as rice, sweet potato or barley. Awamori is one type of *shochu* beverage that is produced from rice. During the production of awamori, rice-koji fermentation starter (*tane*-*koji*) of *A. luchuensis* is used in preparing the rice-koji, and following the completion of fermentation, the alcohol is distilled under ordinary pressure. Awamori *shochu* has a unique aroma profile, and recently, the characteristics of the natural vaporized aroma compounds emitted from awamori were elucidated (Taira et al. 2012). However, the presence of phenolic aroma compounds and the biotransformation have not yet been clarified. Therefore, this study focused on elucidating the biotransformation of phenolic compounds in *A. luchuensis* during fermentation. Consequently, the vanillin production mechanism in the *Aspergillus* species is for the first time proposed based on this study.

## Materials and methods

### Chemicals

Authentic compounds for analysis by mass spectrometry, such as hydroxy-3-methoxycinnamic acid (ferulic acid), 4-hydroxy-3-methoxybenzoic acid (vanillic acid), 4-hydroxy-3-methoxybenzaldehyde (vanillin) and 2-methoxy-4-vinylphenol (4-guaiachol) were commercially available. The rice-koji fermentation starter (*tane koji*) of *A. luchuensis* was obtained from Ishikawa Tanekoji Ten, Ltd. (Okinawa, Japan). Awamori 101 yeast was purchased from Shinzato-shuzo Co., Ltd. (Okinawa, Japan).

### Fermentation and alcohol distillation

Japanese spirit, awamori *shochu* was produced by the general procedures as follows. Briefly, the washed long grain rice (Oryza sativa subsp., indica, 1200 g) was immersed in water for 1 day. It was steamed by an autoclave treatment at 121 °C for 20 min, and it was then cooled to 35 °C. The *tane koji* (10 g) was next added to the steamed rice and its rice-koji was prepared by incubation at 30 °C for 40 h. To the rice-koji (400 g) was added mother water (720 ml) and yeast (awamori 101 yeast, dry weight 0.1 g), then the fermentation was carried out at 26 °C for 15 days. Following the completion of fermentation, a 50% alcohol, except for the first drops for 5 min, was distillated under decompression. Each analytical sample was collected from the rice-koji suspension, the fermentation products at 5, 10, 15 days and the distilled alcohol. The samples of the rice-koji and fermentation product were centrifuged at 5000 rpm for 30 min (HITACH himac CR22GII, Hitachi Koki, Co., Ltd., Tokyo, Japan). All samples were treated by a solid phase extraction step.

### Solid phase extraction

The sample was extracted using a solid phase cartridge column (Oasis HLB Cartridge, Waters). Briefly, the column was conditioned with 5 ml of methanol and MQ-water, then the sample (5 ml) was loaded on the column. The column was continuously washed with 10 ml of MQ-water and the sample was collected with 10 ml of methanol. The solvent was evaporated at room temperature (Centrifugal evaporator CVE-310, Unitraput-1000, EYELA) and the residue dissolved in ethanol (1 ml) was used as the analytical sample.

### LC/MS analysis

Each sample was measured by LC/MS (Agilent1200, Agilent Technologies) using a photodiode array detector and monitored at 280 nm on a reversed-phase chromatographic column, YMC-Pack Pro C18 (100 × 4.6 mm I.D., 5 μm particle size, YMC Co., Ltd., Japan) at 40.0 °C. The mobile phase consisting of a 5 mM formic acid aqueous solution (10%) and acetonitrile was carried out at the flow rate of 0.8 ml/min by a linear gradient to 50% (10 min) and 100% (5 min) and held for 5 min. The mass spectra were measured under the following conditions: ESI negative ion mode; desolvation temperature, 350 °C; desolvation pressure, 35 psig and desolvation gas flow, 12.01 ml/min (6120 Quadrupole, Agilent Technologies).

### LC/MS/MS analysis

The HRESI-mass spectra were measured using an LC/MS/MS (Agilent 6560 IM-QTOF, Agilent Technologies). The LC was then carried out under the same conditions for the LC/MS analysis. The mass spectra were measured under the following conditions: ESI negative ion mode; drying gas temperature, 350 °C; desolvation pressure, 35 psig and desolvation gas flow, 12 ml/min; capillary voltage, 3500 V; nozzle voltage, 500 V; collision, 20 V. Reference ions were used for 19.03632 and 966.000725.

### Bioinformatics

The annotation information of amino acid sequences from *A. luchuensis* genome (accession nos: BCWF01000001–BCWF01000044, Yamada et al. 2016) was searched using BLASTP against the Swiss-Prot and TrEMBL protein databases from Uniprot (e-value cut-off 1e-5). Candidate genes related to vanillin production were searched based on the keywords, such as vanillin, enoyl (feruloyl), glucosidase and glucosyl transferase.

## Results

### Identification of phenolic compounds

**Fig. 1:**
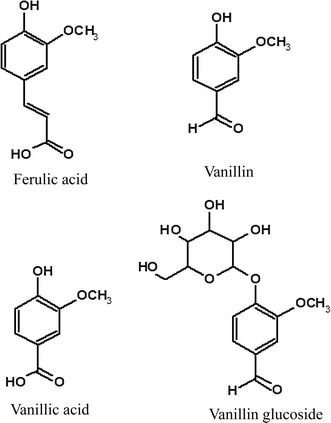
The phenolic compounds were detected in rice-koji fermented by *Aspergillus luchuensis*

**Fig. 2:**
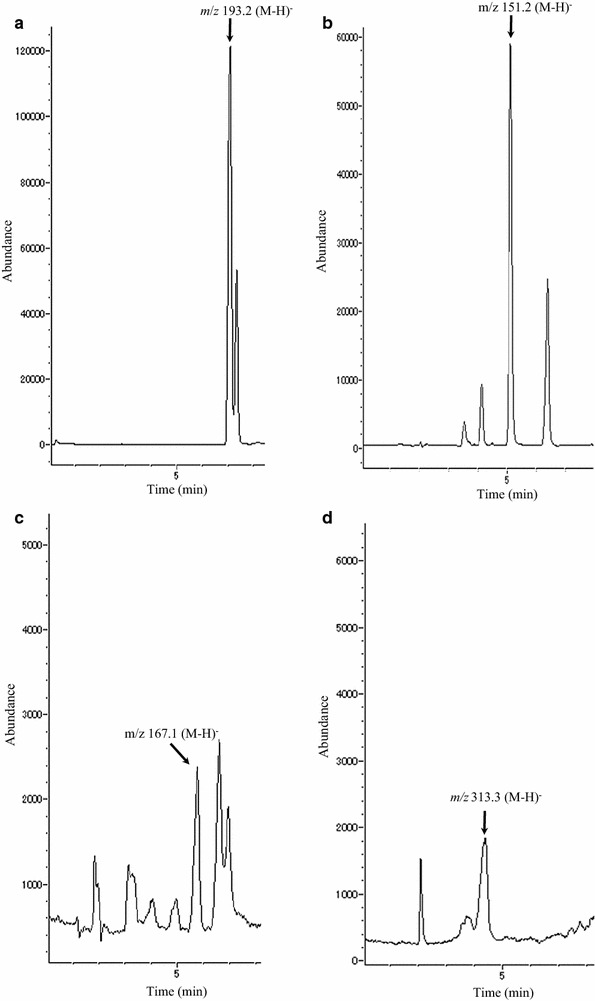
Total ion chromatogram obtained from the biotransformation of phenolic compounds in *Aspergillus luchuensis*. The total ion chromatogram (TIC) was measured by LC/MS. Each TIC mass spectrum for **a** ferulic acid ((M-H)^−^, 193.2), **b** vanillin ((M-H)^−^, 151.2), **c** vanillic acid ((M-H)^−^, 167.1), **d** vanillin glucoside ((M-H)^−^, 313.3) and individual retention times were identical to those of the authentic compounds

**Fig. 3:**
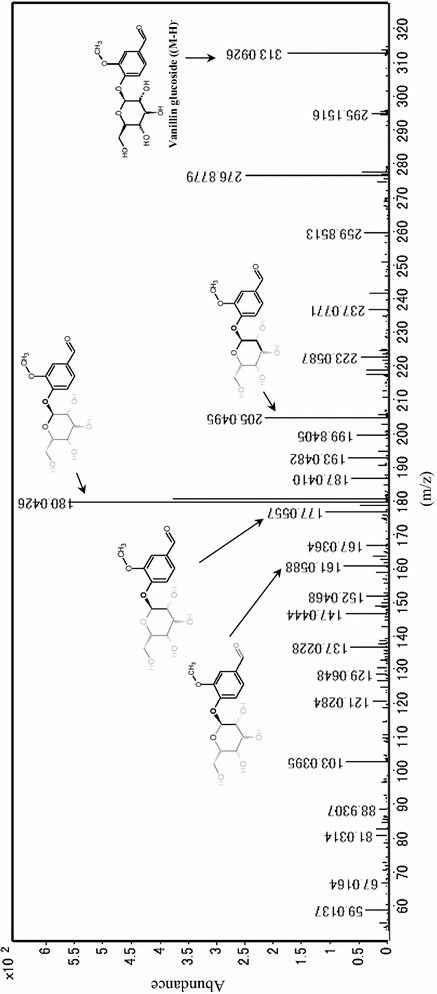
The mass spectrometry of vanillin glucoside produced during the phenolic biotransformation of *Aspergillus luchuensis.* The HRESI-mass spectrometry of vanillin glucoside was measured by LC/MS/MS. The accurate molecular formula of vanillin glucoside was determined to be C_14_H_18_O_8_ based on the mass 313.0927 (M-H)^−^, 313.0926, calcd. for C_14_H_18_O_8_. The other production ions derived from the vanillin (*m/z*: 161.0588, 177.0557, 180.0426 and 205.0495) were also detected which were structurally supported

In this study investigated the phenolic biotransformation including vanillin production due to phenolic biotransformation in *A. luchuensis* during fermentation together with brewing. The phenolic compounds in *A. luchuensis* during fermentation were detected by LC/MS and LC/MS/MS (Fig. 1). Each total ion chromatogram (TIC) of ferulic acid, vanillin, vanillic acid and vanillin glucoside is shown in Fig. 2. The molecular weights and retention time of ferulic acid (193.2, (M-H)^−^, 6.95 min), vanillin (151.2, (M-H)^−^, 4.94 min) and vanillic acid (167.1, (M-H)^−^, 5.89 min) were identical to those of the authentic compounds except for vanillin glucodide. A similar molecular weight ((M-H)^−^, 313.3, 4.33 min) for vanillin glucoside was also obtained from the mash and its fermentation product. The HRESI-mass spectrum of vanillin glucoside was measured to clarify the molecule by LC/MS/MS (Fig. 3). The accurate molecular formula of vanillin glucoside was determined to be C_14_H_18_O_8_ based on the mass of 313.0927 (M-H)^−^, 313.0926, calcd. for C_14_H_18_O_8_. The other production ions derived from the vanillin (*m/z* 161.0588, 177.0557, 180.0426 and 205.0495) were also detected and they were structurally supported to be vanillin glucoside.

### Changes of the phenolic compounds during fermentation

**Fig. 4:**
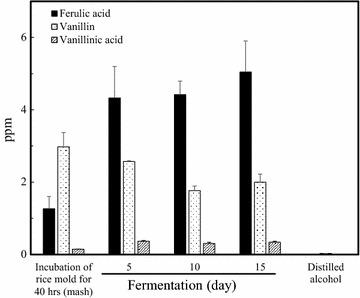
Phenolic compounds of biotransformation in *Aspergillus luchuensis* during fermentation

The contents of these phenolic compounds in the rice mold (rice-koji) without yeast, its fermentation products for 5, 10 and 15 days and the distilled alcohol are indicated in Fig. 4. The phenolic compounds, such as ferulic acid, vanillin and vanillic acid, were detected in the rice-koji and during fermentation, and vanillin and vanillic acid also transferred to the distilled alcohol. These phenolic compounds were produced in the rice-koji and during the fermentation, indicating the metabolites due to phenolic biotransformation in *A. luchuensis*. A previous study reported the detection of phenolic compounds, such as 4-vinyl alcohol, vanillin and vanillic acid are only detected in the distilled awamori *shochu* or its aging (Koseki et al. 1996). Therefore, the phenolic compounds during the biotransformation of *A. luchuensis* were first determined in this study.

Esterified ferulic acid in the cell walls of crops is hydrolyzed by feruloyl esterase. The esterases that the hydrolyzed the ester linkages of ferulic acid in the cell walls have been isolated from *Streptomyces olivochromogenes* (Craig and Williamson 1991), *A. niger* (Craig et al. 1997) and *A. luchuensis* (Koseki et al. 1998). In this study, the ferulic acid was detected in the steam riced materials before the mash, indicating that the ferulic acid is already present in the raw materials (data not shown), then it was accumulated by feruloyl esterase during the fermentation (Fig. 4).

### Bioinformatics for phenolic compounds

The annotation information of amino acid sequences from *A. luchuensis* genome searched using BLAST. Particularly, the related genes to the vanillin production was investigated, and then the possible 82 genes contribute to the phenolic biotransformation were found in the genome information of *A. luchuensis* (Additional file 1: Table S1). Specifically, the genes of feruloyl estelases, enoyl (feruloyl)-CoA synthases, enoyl (feruloyl)-CoA hydratases suggested that they are take part in the vanillin production, and also vanillin dehydrogenase contributes to vanillic acid production due to dehydrogenation of vanillin. Ιn addition, the genes related to glucosidase and glucose transferase suggested another possible pathway to be produced vanillin from vanillin glucoside which was detected in the biotransformation of *A. luchuensis* during fermentation (Fig. 4).

## Discussion

It has been reported that the decarboxylation of ferulic acid due to many lactic acid bacteria strains produces 4-vinyl guaiachol coupled with 4-ethyl phenol and the following oxidation of 4-vinyl guaiachol produced vanillin during the fermentation (Beek and Priest 2000; Huang et al. 1993; Rosazza et al. 1995). In this study, these decarboxylated compounds due to the one carbon elimination of the ferulic acid side chain were confirmed, but it was not detected in any sample. Thus, the vanillin production pathway with decarboxylation will not be suitable for the biotransformation in *A. luchuensis*. Previous articles reported that the elimination of two carbons from the ferulic acid side chain through the hydration and nonoxidative cleavage of feruloyl-CoA (enoyl-CoA) produced vanillin (Narbad and Gasson 1998; Gasson et al. 1998; Overhage et al. 1999). Briefly, the feruloyl-CoA synthetase catalyzes the transfer of CoA to the carboxyl group of ferulic acid, which then forms feruloyl-CoA. The feruloyl-CoA is degraded by the feruloyl-CoA hydratases (enoyl-CoA hydratases)/lyases to form 4-hydroxy-3-methoxyphenyl-hydroxypropionyl-CoA, and its cleavage produces vanillin and acetyl-COA. The produced vanillin from the biotransformation will be dehydrogenated to vanillic acid by vanillin dehydrogenase (Rosazza et al. 1995). The related genes to the vanillin production, such as feruloyl esterase, enoyl-CoA hydratases, vanillin dehydrogenase, glucosidases and glucosyltransferases were detected in the genome information of *A. luchuensis* (Additional file 1: Table S1). The expression genes and related to the enzymes which contribute to the vanillin production pathway will be clarified in our future study.

**Fig. 5:**
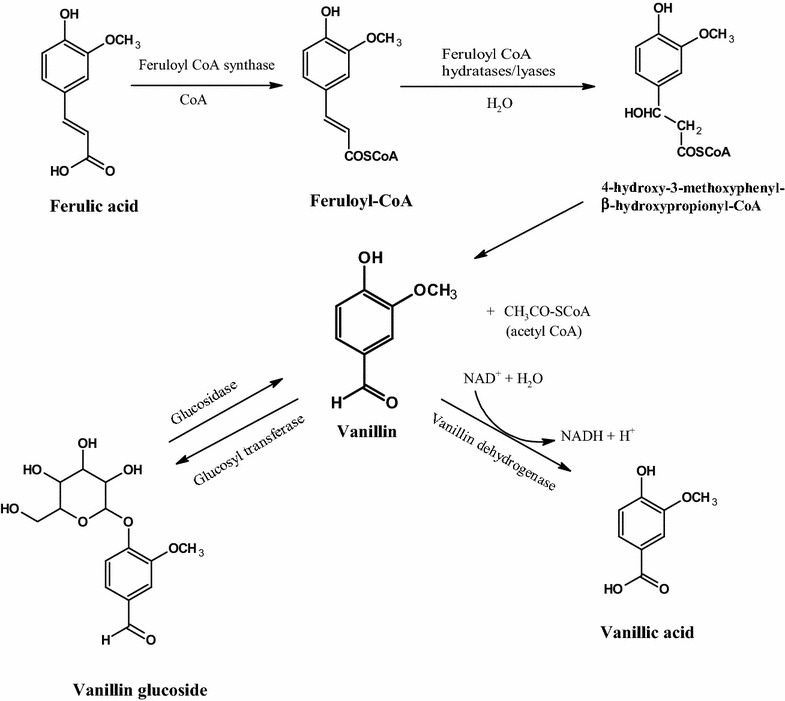
The proposed vanillin production mechanism through phenolic biotransformation in *Aspergillus luchuensis.* The side chain cleavage of ferulic acid through feruloyl-CoA and feruloyl-CoA hydratase/lyase give vanillin and acetyl-COA, then the dehydrogenation of vanillin produced vanillic acid. In addition, the glucose dehydrogenation of the vanillin glucoside is produced vanillin through the reversible reactions

Based on the results including phenolic metabolites and the related genes, this study proposes a similar phenolic biotransformation mechanism that involves in a two-step process, the CoA ligases followed by the side-chain cleavage of ferulic acid through feruloyl-CoA and feruloyl-CoA hydratase/lyase give vanillin and acetyl-COA, then the dehydrogenation of vanillin produced vanillic acid (Fig. 5).

Interestingly, the vanillin glucoside was also first identified in the *Aspergillus* species in the mash and its fermentation. As shown in the vanillin production scheme (Fig. 5), glucose hydrolysis of vanillin glucoside would be produced vanillin through the reversible reactions in the presence of glucosidase and glucosyltransferase (Gallage et al. 2014). This pathway may take part in one of the vanillin production mechanisms. In addition, the vanillin during fermentation was also detected in the distilled alcohol indicating its contribution to the aroma profile of the awamori *shochu.*

In conclusion, this study proposed the phenolic biotransformation mechanism in *A. luchuensis* that is involves a two-step process, i.e., the CoA ligases followed by a two-carbon elimination of ferulic acid produce vanillin and acetyl-COA. Also, another vanillin production pathway derived from the vanillin glucoside was proposed. The following dehydrogenation of vanillin will produce vanillic acid. In addition, vanillin in the distilled alcohol indicated its contribution to the aroma of *shochu*.

## Additional file

**Additional file 1: Table S1.** Candidate genes for vanillin production found in *Aspergillus luchuensis* genome.

## Abbreviations

LC/MS: liquid chromatography–mass spectrometry
ESI: electrospray ionization
LC/MS/MS: liquid chromatography-tandem mass spectrometry
HRESI: high-resolution-electrospray ionization
