# Supplementary material for: Vanillin production by biotransformation of phenolic compounds in fungus, Aspergillus luchuensis
Source: AMB Express. 2018 Mar 13;8:40. doi: 10.1186/s13568-018-0569-4 (PMC5849584; doi:10.1186/s13568-018-0569-4)
Supplement: Supplementary file 1 — Additional file 1: Table S1. Candidate genes for vanillin production found in Aspergillus luchuensis genome. [file 13568_2018_569_MOESM1_ESM.pdf]

Supplementary materials

Journal name: AMB Express

Manuscript Title: Vanillin production by biotransformation of phenolic compounds in fungus, *Aspergillus luchuensis*

The name(s) of the author(s): Junsei Taira (corresponding author), Rin Toyoshima, Nana Ameku, Akira Iguchi, Yasutomo Tamaki

The affiliation(s) and address(es) of the author(s):
Okinawa National College of Technology, Department of Bioresource Technology, 905 Henoko, Nago-city, Okinawa 905-2 192, Japan
The e-mail address, telephone and fax numbers of the corresponding author: Tel & Fax: +81 980-55-4207, E-mail: taira@okinawa-ct.ac.jp

Table S1. Candidate genes for vanillin production found in *Aspergillus luchuensis* genome

Accession number	Annotate description	E-value
BCWF01000001-111	sp|Q5BB53|BGLI_EMENI Probable beta-glucosidase I OS=Emericella nidulans (strain FGSC A4 / ATCC 38163 / CBS 112.46 / NRRL 194 / M139) GN=bglI PE=2 SV=2 (839)	2.00E-66
BCWF01000001-40	sp|A2R0Z6|FAEB_ASPNC Probable feruloyl esterase B OS=Aspergillus niger (strain CBS 513.88 / FGSC A1513) GN=faeB PE=3 SV=1 (521)	0
BCWF01000006-16	sp|Q05871|ECI1_YEAST 3,2-trans-enoyl-CoA isomerase OS=Saccharomyces cerevisiae (strain ATCC 204508 / S288c) GN=ECI1 PE=1 SV=1 (280)	1.00E-36
BCWF01000006-226	sp|Q4WGL5|SUN1_ASPFU Secreted beta-glucosidase sun1 OS=Neosartorya fumigata (strain ATCC MYA-4609 / Af293 / CBS 101355 / FGSC A1100) GN=sun1 PE=1 SV=1 (414)	0
BCWF01000006-280	sp|G4MS28|CEGT_MAGO7 Ceramide glucosyltransferase OS=Magnaporthe oryzae (strain 70-15 / ATCC MYA-4617 / FGSC 8958) GN=MGG_10668 PE=1 SV=1 (494)	3.00E-158
BCWF01000006-381	sp|A2QN74|BTGC_ASPNC Putative glucan endo-1,3-beta-glucosidase btgC OS=Aspergillus niger (strain CBS 513.88 / FGSC A1513) GN=btgC PE=3 SV=2 (684)	0
BCWF01000006-528	sp|Q09140|UGGG_SCHPO UDP-glucose:glycoprotein glucosyltransferase OS=Schizosaccharomyces pombe (strain 972 / ATCC 24843) GN=gpt1 PE=1 SV=2 (1448)	0
BCWF01000006-535	sp|P27034|BGLS_RHIRD Beta-glucosidase OS=Rhizobium radiobacter GN=cbg-1 PE=3 SV=1 (818)	7.00E-114
BCWF01000006-551	sp|A2QNQ5|ATG26_ASPNC Sterol 3-beta-glucosyltransferase OS=Aspergillus niger (strain CBS 513.88 / FGSC A1513) GN=atg26 PE=3 SV=2 (1371)	0
BCWF01000006-829	sp|A2QPK4|BGLD_ASPNC Probable beta-glucosidase D OS=Aspergillus niger (strain CBS 513.88 / FGSC A1513) GN=bglD PE=3 SV=2 (754)	0
BCWF01000006-97	sp|P49426|EXG1_COCCA Glucan 1,3-beta-glucosidase OS=Cochliobolus carbonum GN=EXG1 PE=1 SV=1 (788)	4.00E-126
BCWF01000008-185	sp|O35459|ECH1_MOUSE Delta(3,5)-Delta(2,4)-dienoyl-CoA isomerase, mitochondrial OS=Mus musculus GN=Ech1 PE=1 SV=1 (327)	2.00E-74
BCWF01000008-195	sp|Q9FZE0|BGL40_ARATH Beta-glucosidase 40 OS=Arabidopsis thaliana GN=BGLU40 PE=2 SV=1 (510)	3.00E-47
BCWF01000009-8	sp|D4B0V1|E13B_ARTBC Probable glucan endo-1,3-beta-glucosidase ARB_02077 OS=Arthroderma benhamiae (strain ATCC MYA-4681 / CBS 112371) GN=ARB_02077 PE=1 SV=1 (878)	4.00E-102
BCWF01000009-95	sp|D4AS70|FAE1_ARTBC Probable feruloyl esterase ARB_07085 OS=Arthroderma benhamiae (strain ATCC MYA-4681 / CBS 112371) GN=ARB_07085 PE=1 SV=1 (537)	4.00E-89
BCWF01000010-127	sp|D4AS70|FAE1_ARTBC Probable feruloyl esterase ARB_07085 OS=Arthroderma benhamiae (strain ATCC MYA-4681 / CBS 112371) GN=ARB_07085 PE=1 SV=1 (537)	3.00E-122
BCWF01000010-325	sp|O74799|EXG3_SCHPO Glucan 1,3-beta-glucosidase 3 OS=Schizosaccharomyces pombe (strain 972 / ATCC 24843) GN=exg3 PE=3 SV=1 (464)	9.00E-08
BCWF01000010-343	sp|P87076|BGLA_ASPKW Beta-glucosidase A OS=Aspergillus kawachii (strain NBRC 4308) GN=bglA PE=1 SV=2 (860)	0
BCWF01000010-391	sp|O05619|VDH_PSEUH Vanillin dehydrogenase OS=Pseudomonas sp. (strain HR199 / DSM 7063) GN=vdh PE=1 SV=1 (481)	2.00E-100
BCWF01000010-395	sp|A2RAR6|EXGA_ASPNC Probable glucan 1,3-beta-glucosidase A OS=Aspergillus niger (strain CBS 513.88 / FGSC A1513) GN=exgA PE=3 SV=1 (416)	0
BCWF01000010-529	sp|Q9F234|AGL2_BACTQ Alpha-glucosidase 2 OS=Bacillus thermoamyloliquefaciens PE=3 SV=1 (787)	2.00E-52
BCWF01000015-126	sp|D4AS70|FAE1_ARTBC Probable feruloyl esterase ARB_07085 OS=Arthroderma benhamiae (strain ATCC MYA-4681 / CBS 112371) GN=ARB_07085 PE=1 SV=1 (537)	6.00E-96
BCWF01000015-37	sp|A5ABF5|BGLM_ASPNC Probable beta-glucosidase M OS=Aspergillus niger (strain CBS 513.88 / FGSC A1513) GN=bglM PE=3 SV=1 (765)	0
BCWF01000015-401	sp|Q05871|ECI1_YEAST 3,2-trans-enoyl-CoA isomerase OS=Saccharomyces cerevisiae (strain ATCC 204508 / S288c) GN=ECI1 PE=1 SV=1 (280)	3.00E-40
BCWF01000015-590	sp|Q2UN12|BGLF_ASPOR Probable beta-glucosidase F OS=Aspergillus oryzae (strain ATCC 42149 / RIB 40) GN=bglF PE=3 SV=1 (866)	0
BCWF01000015-77	sp|Q9XIG1|U80B1_ARATH Sterol 3-beta-glucosyltransferase UGT80B1 OS=Arabidopsis thaliana GN=UGT80B1 PE=2 SV=1 (615)	2.00E-09
BCWF01000015-78	sp|Q9XIG1|U80B1_ARATH Sterol 3-beta-glucosyltransferase UGT80B1 OS=Arabidopsis thaliana GN=UGT80B1 PE=2 SV=1 (615)	3.00E-77
BCWF01000016-15	sp|P27034|BGLS_RHIRD Beta-glucosidase OS=Rhizobium radiobacter GN=cbg-1 PE=3 SV=1 (818)	1.00E-169
BCWF01000017-1081	sp|O43053|ALG6_SCHPO Probable dolichyl pyrophosphate Man9GlcNAc2 alpha-1,3-glucosyltransferase OS=Schizosaccharomyces pombe (strain 972 / ATCC 24843) GN=alg6 PE=3 SV=1 (506)	5.00E-133
BCWF01000017-1129	sp|P53626|E13B_TRIHA Glucan endo-1,3-beta-glucosidase BGN13.1 OS=Trichoderma harzianum GN=bgn13.1 PE=1 SV=1 (762)	1.00E-90
BCWF01000017-1136	sp|Q9P6J3|MALT_SCHPO Alpha-glucosidase OS=Schizosaccharomyces pombe (strain 972 / ATCC 24843) GN=mal1 PE=2 SV=1 (579)	0
BCWF01000017-346	sp|D4B0V1|E13B_ARTBC Probable glucan endo-1,3-beta-glucosidase ARB_02077 OS=Arthroderma benhamiae (strain ATCC MYA-4681 / CBS 112371) GN=ARB_02077 PE=1 SV=1 (878)	0
BCWF01000017-438	sp|D4AS70|FAE1_ARTBC Probable feruloyl esterase ARB_07085 OS=Arthroderma benhamiae (strain ATCC MYA-4681 / CBS 112371) GN=ARB_07085 PE=1 SV=1 (537)	2.00E-93
BCWF01000017-501	sp|Q4WRH9|AGDC_ASPFU Probable alpha/beta-glucosidase agdC OS=Neosartorya fumigata (strain ATCC MYA-4609 / Af293 / CBS 101355 / FGSC A1100) GN=agdC PE=3 SV=1 (881)	0
BCWF01000017-604	sp|F4JML5|ECH2M_ARATH Probable enoyl-CoA hydratase 2, mitochondrial OS=Arabidopsis thaliana GN=At4g16800 PE=2 SV=1 (301)	2.00E-54
BCWF01000017-681	sp|Q9XIG1|U80B1_ARATH Sterol 3-beta-glucosyltransferase UGT80B1 OS=Arabidopsis thaliana GN=UGT80B1 PE=2 SV=1 (615)	9.00E-13
BCWF01000017-880	sp|G3V9D0|PGLT1_RAT Protein O-glucosyltransferase 1 OS=Rattus norvegicus GN=Poglut1 PE=3 SV=1 (392)	6.00E-09
BCWF01000018-649	sp|A2QPK4|BGLD_ASPNC Probable beta-glucosidase D OS=Aspergillus niger (strain CBS 513.88 / FGSC A1513) GN=bglD PE=3 SV=2 (754)	8.00E-36
BCWF01000018-75	sp|Q0CBM7|FAEA_ASPTN Probable feruloyl esterase A OS=Aspergillus terreus (strain NIH 2624 / FGSC A1156) GN=faeA PE=3 SV=1 (281)	2.00E-20
BCWF01000019-184	sp|A5ABF5|BGLM_ASPNC Probable beta-glucosidase M OS=Aspergillus niger (strain CBS 513.88 / FGSC A1513) GN=bglM PE=3 SV=1 (765)	0
BCWF01000020-280	sp|Q2UP89|FAEB1_ASPOR Probable feruloyl esterase B-1 OS=Aspergillus oryzae (strain ATCC 42149 / RIB 40) GN=faeB-1 PE=1 SV=1 (540)	6.00E-92
BCWF01000020-313	sp|Q25BW5|BGL1A_PHACH Beta-glucosidase 1A OS=Phanerochaete chrysosporium GN=BGL1A PE=1 SV=1 (462)	1.00E-53

BCWF01000020-519	sp|A2QPK4|BGLD_ASPNC Probable beta-glucosidase D OS=Aspergillus niger (strain CBS 513.88 / FGSC A1513) GN=bglD PE=3 SV=2 (754)	0
BCWF01000020-549	sp|O94511|YN67_SCHPO Putative enoyl reductase C646.07c OS=Schizosaccharomyces pombe (strain 972 / ATCC 24843) GN=SPBC646.07c PE=3 SV=1 (295)	3.00E-71
BCWF01000020-629	sp|P56526|AGLU_ASPNG Alpha-glucosidase OS=Aspergillus niger GN=aglA PE=1 SV=1 (985)	0
BCWF01000020-760	sp|Q2UB20|ALG8_ASPOR Dolichyl pyrophosphate Glc1Man9GlcNAc2 alpha-1,3-glucosyltransferase OS=Aspergillus oryzae (strain ATCC 42149 / RIB 40) GN=alg8 PE=3 SV=1 (504)	0
BCWF01000020-776	sp|Q7D745|Y2525_MYCTO Putative trans-acting enoyl reductase MT2525 OS=Mycobacterium tuberculosis (strain CDC 1551 / Oshkosh) GN=MT2525 PE=3 SV=2 (419)	1.00E-31
BCWF01000021-150	sp|Q1ZXF1|ECHM_DICDI Probable enoyl-CoA hydratase, mitochondrial OS=Dictyostelium discoideum GN=echs1 PE=3 SV=1 (277)	3.00E-101
BCWF01000021-170	sp|Q4X162|ALG10_ASPFU Dol-P-Glc:Glc(2)Man(9)GlcNAc(2)-PP-Dol alpha-1,2-glucosyltransferase OS=Neosartorya fumigata (strain ATCC MYA-4609 / Af293 / CBS 101355 / FGSC A1100) GN=alg10 PE=3 SV=1	0
BCWF01000021-231	sp|P96559|GTFB_AMYOR Vancomycin aglycone glucosyltransferase OS=Amycolatopsis orientalis GN=gtfB PE=1 SV=1 (407)	1.00E-06
BCWF01000021-992	sp|Q01336|YCR2_ESCVU Uncharacterized family 31 glucosidase ORF2 (Fragment) OS=Escherichia vulneris PE=3 SV=1 (529)	0
BCWF01000023-149	sp|Q2UP89|FAEB1_ASPOR Probable feruloyl esterase B-1 OS=Aspergillus oryzae (strain ATCC 42149 / RIB 40) GN=faeB-1 PE=1 SV=1 (540)	7.00E-92
BCWF01000023-178	sp|O05619|VDH_PSEUH Vanillin dehydrogenase OS=Pseudomonas sp. (strain HR199 / DSM 7063) GN=vdh PE=1 SV=1 (481)	5.00E-75
BCWF01000024-102	sp|A2QX52|EXGD_ASPNC Probable glucan 1,3-beta-glucosidase D OS=Aspergillus niger (strain CBS 513.88 / FGSC A1513) GN=exgD PE=3 SV=1 (830)	0
BCWF01000025-366	sp|Q2UP89|FAEB1_ASPOR Probable feruloyl esterase B-1 OS=Aspergillus oryzae (strain ATCC 42149 / RIB 40) GN=faeB-1 PE=1 SV=1 (540)	4.00E-67
BCWF01000026-16	sp|O05619|VDH_PSEUH Vanillin dehydrogenase OS=Pseudomonas sp. (strain HR199 / DSM 7063) GN=vdh PE=1 SV=1 (481)	3.00E-108
BCWF01000026-7	sp|Q2US39|BTGE_ASPOR Probable beta-glucosidase btgE OS=Aspergillus oryzae (strain ATCC 42149 / RIB 40) GN=btgE PE=3 SV=1 (602)	0
BCWF01000028-305	sp|D4B2W4|BGL2_ARTBC Glucan 1,3-beta-glucosidase ARB_02797 OS=Arthroderma benhamiae (strain ATCC MYA-4681 / CBS 112371) GN=ARB_02797 PE=1 SV=1 (308)	5.00E-121
BCWF01000028-86	sp|A2RAR6|EXGA_ASPNC Probable glucan 1,3-beta-glucosidase A OS=Aspergillus niger (strain CBS 513.88 / FGSC A1513) GN=exgA PE=3 SV=1 (416)	3.00E-25
BCWF01000029-114	sp|Q2UP89|FAEB1_ASPOR Probable feruloyl esterase B-1 OS=Aspergillus oryzae (strain ATCC 42149 / RIB 40) GN=faeB-1 PE=1 SV=1 (540)	2.00E-19
BCWF01000029-134	sp|Q2UTX5|BGLE_ASPOR Probable beta-glucosidase E OS=Aspergillus oryzae (strain ATCC 42149 / RIB 40) GN=bglE PE=3 SV=2 (1048)	0
BCWF01000029-177	sp|O14255|GCS1_SCHPO Probable mannosyl-oligosaccharide glucosidase OS=Schizosaccharomyces pombe (strain 972 / ATCC 24843) GN=SPAC6G10.09 PE=3 SV=1 (808)	0
BCWF01000029-193	sp|Q05871|ECI1_YEAST 3,2-trans-enoyl-CoA isomerase OS=Saccharomyces cerevisiae (strain ATCC 204508 / S288c) GN=ECI1 PE=1 SV=1 (280)	9.00E-54
BCWF01000029-43	sp|P32573|SPS19_YEAST Peroxisomal 2,4-dienoyl-CoA reductase SPS19 OS=Saccharomyces cerevisiae (strain ATCC 204508 / S288c) GN=SPS19 PE=1 SV=4 (292)	7.00E-84
BCWF01000030-146	sp|A2QH21|EGLC_ASPNC Probable glucan endo-1,3-beta-glucosidase eglC OS=Aspergillus niger (strain CBS 513.88 / FGSC A1513) GN=eglC PE=3 SV=2 (460)	0
BCWF01000030-155	sp|A1DFA8|BGLI_NEOFI Probable beta-glucosidase I OS=Neosartorya fischeri (strain ATCC 1020 / DSM 3700 / CBS 544.65 / FGSC A1164 / JCM 1740 / NRRL 181 / WB 181) GN=bglI PE=3 SV=1 (838)	0
BCWF01000030-3	sp|Q25BW4|BGL1B_PHACH Beta-glucosidase 1B OS=Phanerochaete chrysosporium GN=BGL1B PE=1 SV=1 (540)	0
BCWF01000030-54	sp|P40350|ALG5_YEAST Dolichyl-phosphate beta-glucosyltransferase OS=Saccharomyces cerevisiae (strain ATCC 204508 / S288c) GN=ALG5 PE=1 SV=1 (334)	2.00E-75
BCWF01000032-211	sp|Q2UP89|FAEB1_ASPOR Probable feruloyl esterase B-1 OS=Aspergillus oryzae (strain ATCC 42149 / RIB 40) GN=faeB-1 PE=1 SV=1 (540)	1.00E-80
BCWF01000032-49	sp|D4AS70|FAE1_ARTBC Probable feruloyl esterase ARB_07085 OS=Arthroderma benhamiae (strain ATCC MYA-4681 / CBS 112371) GN=ARB_07085 PE=1 SV=1 (537)	3.00E-33
BCWF01000033-14	sp|Q6CBE4|ETR1_YARLI Probable trans-2-enoyl-CoA reductase, mitochondrial OS=Yarrowia lipolytica (strain CLIB 122 / E 150) GN=ETR1 PE=3 SV=1 (376)	7.00E-93
BCWF01000034-14	sp|Q56078|BGLX_SALTY Periplasmic beta-glucosidase OS=Salmonella typhimurium (strain LT2 / SGSC1412 / ATCC 700720) GN=bglX PE=3 SV=2 (765)	3.00E-115
BCWF01000034-31	sp|A2R989|BGLI_ASPNC Probable beta-glucosidase I OS=Aspergillus niger (strain CBS 513.88 / FGSC A1513) GN=bglI PE=3 SV=1 (818)	0
BCWF01000036-120	sp|O13716|AGN1_SCHPO Glucan endo-1,3-alpha-glucosidase agn1 OS=Schizosaccharomyces pombe (strain 972 / ATCC 24843) GN=agn1 PE=1 SV=2 (424)	1.00E-50
BCWF01000036-126	sp|Q5B0F4|BGLG_EMENI Probable beta-glucosidase G OS=Emericella nidulans (strain FGSC A4 / ATCC 38163 / CBS 112.46 / NRRL 194 / M139) GN=bglG PE=3 SV=2 (819)	0
BCWF01000036-127	sp|Q2U325|BGLG_ASPOR Probable beta-glucosidase G OS=Aspergillus oryzae (strain ATCC 42149 / RIB 40) GN=bglG PE=3 SV=1 (815)	0
BCWF01000036-134	sp|Q9XIG1|U80B1_ARATH Sterol 3-beta-glucosyltransferase UGT80B1 OS=Arabidopsis thaliana GN=UGT80B1 PE=2 SV=1 (615)	7.00E-126
BCWF01000036-145	sp|D4AS70|FAE1_ARTBC Probable feruloyl esterase ARB_07085 OS=Arthroderma benhamiae (strain ATCC MYA-4681 / CBS 112371) GN=ARB_07085 PE=1 SV=1 (537)	7.00E-10
BCWF01000036-163	sp|Q2UP89|FAEB1_ASPOR Probable feruloyl esterase B-1 OS=Aspergillus oryzae (strain ATCC 42149 / RIB 40) GN=faeB-1 PE=1 SV=1 (540)	1.00E-72
BCWF01000036-45	sp|O05619|VDH_PSEUH Vanillin dehydrogenase OS=Pseudomonas sp. (strain HR199 / DSM 7063) GN=vdh PE=1 SV=1 (481)	2.00E-100
BCWF01000036-8	sp|B0X1Q4|RUMI_CULQU O-glucosyltransferase rumi homolog OS=Culex quinquefasciatus GN=CPIJ013394 PE=3 SV=1 (403)	2.00E-11
BCWF01000040-41	sp|Q9P6J3|MALT_SCHPO Alpha-glucosidase OS=Schizosaccharomyces pombe (strain 972 / ATCC 24843) GN=mal1 PE=2 SV=1 (579)	2.00E-149
